# Dephosphorylated NSSR1 Is Induced by Androgen in Mouse Epididymis and Phosphorylated NSSR1 Is Increased during Sperm Maturation

**DOI:** 10.1371/journal.pone.0025667

**Published:** 2011-09-29

**Authors:** Ping-Jie Xiao, Zheng-Yu Peng, Lu Huang, Ya Li, Xian-Hua Chen

**Affiliations:** 1 Laboratory of Genomic Physiology and State Key Laboratory of Medical Neurobiology, Institutes of Brain Science, Fudan University, Shanghai, China; 2 Institutes of Biomedical Sciences, Fudan University, Shanghai, China; 3 School of Life Sciences, Fudan University, Shanghai, China; St. Georges University of London, United Kingdom

## Abstract

NSSR1 (Neural salient serine/arginine rich protein 1, alternatively SRp38) is a newly identified RNA splicing factor and predominantly expressed in neural tissues. Here, by Western blot analysis and immunofluorescent staining, we showed that the expression of dephosphorylated NSSR1 increased significantly during development of the caput epididymis. In adult mice, phosphorylated NSSR1 was mainly expressed in the apical side of epithelial cells, and dephosphorylated NSSR1 in caput epididymis was upregulated in a testosterone dependent manner. In addition, subcellular immunoreactive distribution of NSSR1 varied in different regions of the epididymis. With respect to the sperm, phosphorylated NSSR1 was detected in the mid-piece of the tail as well as the acrosome. Furthermore, NSSR1 was released from the sperm head during the capacitation and acrosome reaction. These findings for the first time provide the evidence for the potential roles of NSSR1 in sperm maturation and fertilization.

## Introduction

Alternative pre-mRNA splicing is emerging as an important mechanism of genetic diversity [1], [2], [3]. Recent microarray data show that 74% of human genes undergo alternative splicing, which generates different protein isoforms [2]. The splicing is processed in the spliceosome composed of five small nuclear ribonucleoprotein particles (snRNPs: U1, U2, U4/U6, and U5) and various non-snRNP proteins [4]. Serine/arginine rich (SR) proteins, as the major components of non-snRNP proteins, are critical for the selective splicing and highly regulated in various physiologic and pathologic conditions [5]. In addition to the classic pre-mRNA splicing that was processed in the nucleus, the SR proteins may also participate in the minor class of pre-mRNA splicing in the cytoplasm [6], [7], although contradictory evidences still exist [8].

Neural salient serine/arginine rich protein (NSSR) is a newly reported SR protein originally identified in human leukemia cell lines as translocation liposarcoma protein (TLS)-associated SR proteins and has two isoforms (NSSR1 and NSSR2) [9], [10]. It has been shown that dephosphorylated NSSR1 functions as a splicing repressor and is required for global inhibition of splicing both in M phase of the cell cycle and following heat shock by disrupting the association of snRNPs and pre-mRNAs and preventing spliceosome assembly [11], [12]. On the contrary, phosphorylated NSSR1 has been shown to be able to induce formation of spliceosomal complex A in a cell extract lacking SR proteins and functions as a sequence-specific splicing activator [13]. Alternative splicing of many genes such as AMPA receptor subunit GluR-B [10], Low-density lipoprotein receptor (LDLR) [14] and Tyrosine Kinase Receptor C (Trk C) [15] have been found to be regulated by NSSR1. Furthermore, NSSR1 regulates cardiac-specific alternative splicing of triadin pre-mRNA and is required for Ca2+ handling during embryonic heart development [9]. However, the function and molecular mechanism of NSSR1 in alternative splicing is far from elucidated.

NSSR1 is predominantly expressed in neural tissues such as cerebral neurons, cerebellar Purkinje cells and retinal bipolar cells, and plays important roles in regulating neural differentiation and neural-specific alternative splicing [16], [17]. In addition, recently we have reported that NSSR1 is also highly expressed in reproductive system, indicating the potential roles NSSR1 may play in reproduction [18]. In mouse female reproductive system, we found that NSSR1 is developmentally expressed in the uterus and extensively distributed in endometrial carcinoma [19]. In male reproductive system, our results showed that the expression of testicular NSSR1 increased significantly during mouse testes development and NSSR1 was mainly expressed in germ cells, but barely detected in sertoli cells. Testicular NSSR1 was mostly phosphorylated and cytosolic in germ cells [18]. The findings indicate that NSSR1 may be involved in sperm maturation and fertilization.

Because spermatozoa are synthetically inactive, sperm maturation involves the interaction of spermatozoa with proteins that are synthesized and secreted in a region-dependent manner from the epididymal epithelium. It is only during transit through the epididymis that spermatozoa undergo maturation and acquire progressive motility and the ability to fertilize ova [20]. However, despite considerable effort, the molecular and biochemical events that are integral for epididymal sperm maturation are unknown.

The present study was designed to characterize the expression and distribution of NSSR1 in mouse epididymis and to test if the expression of NSSR1 is regulated by hormones such as androgen and estradiol. The expression of NSSR1 during the maturation of mouse sperm and acrosome reaction was also investigated. These findings for the first time provide the evidence for the potential roles of NSSR1 in biological functions of the sperm.

## Materials and Methods

### Experimental animals and treatment

This study was carried out in strict accordance with the recommendations in the Guide for the Care and Use of Laboratory Animals of the National Institutes of Health. All mouse care and experimental were approved by the Institutional Animal Care and Use Committee of Fudan University Shanghai Medical College (IACUC Animal Project Number: 20070116-xu). All surgery was performed under sodium pentobarbital anesthesia, and all efforts were made to minimize suffering.

Mouse epididymal tissues were prepared for the protein extraction or fixed by 4% paraformaldehyde/PBS as described previously [21]. The surgery of unilateral-castrated or bilateral castrated animals was performed as described previously [22]. In brief, after mice were intraperitoneally anesthetized with 0.1 mL/100 g soluble pentobarbitone (30 g/L), a longitudinal incision was made in the midline of the scrotal region, and one of the testes was exposed. Next, the vascular pedicle was ligated and sectioned. After removal of the testis, the skin was sutured with No. 4 silk thread. The mice in the sham operation group were subject to the surgery procedure as the same as in the castrated group but no testis was resected. One week after surgery, mice (9 mice/group) were intraperitoneally injected with testosterone (0.125 mg or 0.5 mg, Sigma, St Louis, USA.) alone, testosterone plus flutamide (0.125 mg/1 mg, Sigma), or with 0.2 mg estradiol for 5 days before sacrifice. Mice in the control were intraperitoneally injected with 50 µl sesame oil in the sham operated and castrated animal groups. The harvested epididymis were stored at −70°C temporally for protein extraction or fixed by 4% paraformaldehyde/PBS.

Sperm cells were collected following the reported method [23]. Briefly, initial segment, caput, corpus and cauda epididymidis was identified and excised into 2 mm incisions and then rinsed with medium containing 150 mM NaCl, 5 mM KCl, 2 mM CaCl_2_,1 mM MgCl_2_, 30 mM HEPES, 10 mM glucose, 10 mM lactic acid, and 1 mM pyruvic acid (pH 7.4). After transfer to 10 ml of medium supplemented with 5 mg of bovine serum albumin per ml and 15 mM NaHCO_3_ individually, semen was allowed to exude (15 min at 37°C, 5% CO_2_) from small incisions. Cells were centrifuged (1000 g, 5 min) and washed by PBS for 2 times and then used for Western blot analysis or immunofluorescent staining, or for acrosome reaction. To load same amount of sperm lysates for Western blot analysis, the number of sperm cells was determined by hemocytometer and β-actin was used as the loading control. Fractionation of heads and tails of the sperms were performed according to the report [24]. In brief, sperms were gently suspended in PBS containing 1 mM phenylmethylsulfonyl fluoride (PBS-PMSF) and sonicated at 30-s intervals using a sonifier (Soniprep 150, SANYO). The resulting mixture of head and tail was pelleted at 600 *g* at 4°C, resuspended in 65% sucrose in PBS-PMSF, and centrifuged through a sucrose step gradient (75, 70, 65% (w/v)) at 104,000 *g* at 4°C for 60 min. The head fraction was collected as a pellet. The tail-rich fraction at the 65–70% sucrose interface was collected, resuspended, and purified further by centrifugation (104,000 *g*, 4°C, 60 min) on a 65–75% sucrose gradient. The fractions of head and tail were verified by light microscopy. Cauda epididymal spermatozoa were used for acrosome reaction. Briefly, spermatozoa were diluted to a concentration of 10^6^ motile spermatozoa/ml in Whitten's media [25], after incubated for 2 hours at 37°C in an incubator supplied with 5% CO_2_, spermatozoa were incubated with A23187 (Sigma) for 1 h to fulfill the acrosome reaction. A 5 mM A23187 stock solution in dimethyl sulphoxide (DMSO) was diluted by 10 times with serum-free HTF medium before a small volume was added to the sperm suspension to achieve the required final concentration of A23187 [26]. Similar dilution was made for the DMSO control solution. Spermatozoa from the same donor were used as controls and incubated with the same medium containing equivalent concentrations of DMSO. After the acrosome reaction, the sperm suspensions were collected and used for Western blot analysis or immunofluorescent staining.

### Western blot analysis

The generation of rabbit anti-NSSR1 antibodies (primary antibodies) and procedure for Western blotting were described previously. In the previous publication, the specificity of the antibody to NSSR1 protein was demonstrated by Western blotting and the pre-absorption of the antibodies with the His-tagged NSSR1 protein [17]. For Western blotting, protein lysates extracted from mouse epididymis, sperms, or head and tail of sperms, were separated by 10% SDS-PAGE (SDS Polyacrylamide Gel Electrophoresis) and transferred to nitrocellulose membranes. AP (alkaline phosphatase)-conjugated goat anti-rabbit IgG antibody (1∶800, Sigma) was used as secondary antibody. The signals were developed by BCIP/NBT (5-bromo-4-chloro-3 -indolyl phosphate/nitroblue tetrazolium) substrates.

### Immunofluorescent staining

Mouse epididymis sections were post-fixed with 4% PFA for 15 min at room temperature. Sperm cells were fixed 4% PFA on glass slide and air dried at room temperature, and then treated with MeOH at −20°C for 30 min. Immunofluorescent labeling was performed following the standard method with rabbit anti-NSSR1 antiserum as primary antibodies and FITC-labeled goat anti-rabbit IgG as secondary antibodies [16]. The specificity of anti-NSSR1 antibodies has been demonstrated previously [17]. The nuclei were stained with DAPI. Signals were detected using a Leica Fluorescence microscope. The color of DAPI (blue) and FITC (green) in the images have been presented with cyan and red, respectively to improve the quality of the merged images.

### Pre-absorption of the NSSR1 antibody

NSSR1 specific peptide (RGTSKTDSKTHYKSC) which was previously used [17] as the immunizing antigen was used to incubate with the antibody at 4°C for overnight, and centrifugated at 13,000 rpm for 10 min before the supernatant was used for Western blot or immunofluorescent staining. A non-specific peptide (CKAVDDITKKQKSKF) was used as the control.

### Statistical study

Experiments were performed in triplicates and repeated at least three times independently. The data were presented as M±SE and corresponding significance was examined by Student's test.

## Results

### NSSR1 expression in mouse epididymis

**Figure 1:**
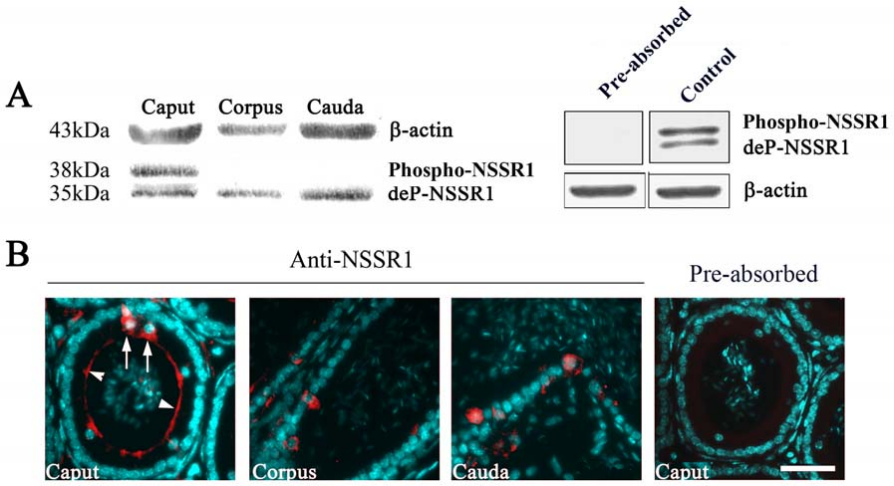
Representative images showing the regional expression of NSSR1 in mouse epididymis. A. Western blot analysis of NSSR1 in three main regions of epididymis: caput, corpus and cauda. The bands detected in epididymis caput were blocked by pre-absorption of the antiserum with specific NSSR1 peptide (Pre-absorbed), but not with non-specific peptide (Contol) (The right panel). B. Representative images showing the immunofluorescent staining of NSSR1 (presented in red) and nuclei (presented in cyan) in different regions of mouse epididymis. Pre-absorption of the antiserum with specific NSSR1 peptide block the immunofluorescent staining (The right panel). Arrows show the principle cells and arrowheads show the apical side of epididymal epithelium. Bar = 40 µm.

In the previous study, we have shown that NSSR1 is regulated in testes development and cryptorchidism and promotes the exon 5-included splicing of CREB transcripts, suggesting the potential role of NSSR1 in spermatogenesis and cryptorchidism. We found that the phosphorylated (38 KDa) and dephosphorylated (35 KDa) NSSR1 were expressed in testes by CIP treatment and Western blot analysis [18]. In order to determine the role of NSSR1 during sperm maturation, we first examined the spatial pattern of NSSR1 expression in matured epididymis. By Western blot, both phosphorylated and dephosphorylated NSSR1 were detected in the adult (8W) epididymis caput, after pre-absorption of the NSSR1 antibody with the specific NSSR1 peptide, these two bands disappeared (Fig. 1A, right panel). In the caput, both phosphorylated and de-phosphorylated NSSR1 proteins were detected, while in the corpus and cauda, only de-phosporylated NSSR1 was detected (Fig. 1A, left panel). The immunofluorescent staining analysis detected the distribution of NSSR1 in the apical side of epithelial cells and the principle cells of epididymis, which can be blocked by pre-absorption of the antibody with the specific NSSR1 peptide (Fig.1B). As the epithelial cells and the principle cells play important roles in the sperm maturation in the epididymis (Fig. 1B), this result indicates that the NSSR1 may be involved in sperm maturation.

**Figure 2:**
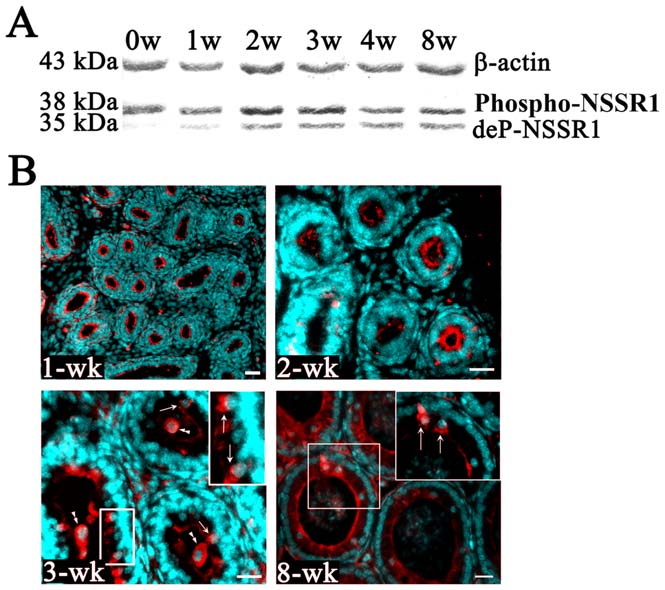
NSSR1 expression in mouse caput epdidymis at various developmental stages. A. Western blot analysis of NSSR1 in mouse caput epididymis at 0-, 1-, 2-, 3-, 4-, 8-week (w). B. Representative immunohistological images showing NSSR1 (red) in the 1-, 2-, 3- and 8-wk caput epididymis with nuclear stain (cyan). Arrows show the principle cells and double arrowheads show the testicular germ cells. Bars = 20 µm.

We next examined the regulation of NSSR1 expression during epididymis development, by characterizing the caput NSSR1 within 8 weeks post-natal using Western blot and IHC analyses. The level of de-phosphorylated NSSR1 proteins in the caput region was up-regulated during the first 3 weeks after born, and remained stable afterwards, while phosphorylated NSSR1 was constantly highly expressed from newborn to adult (Fig. 2A). In addition, we examined the distribution of NSSR1 in the epididymis of 1-wk, 2-wk, 3-wk and 8-wk old mice from the same litter. We found that the signal of NSSR1 immunostaining was both in the lumen and principle cells of the caput epididymis of 3-wk and 8-wk old mice, but only in the lumen of the caput epididymis (alternatively the apical side of epithelial cells) in 0–3 weeks old mice (Fig. 2B). These results indicate that the de-phosphorylated NSSR1 proteins might play important roles in the development of epididymis.

### De-phosphorylated NSSR1 is hormonally regulated in the caput epididymis and primary cultured epididymal epithelium

**Figure 3:**
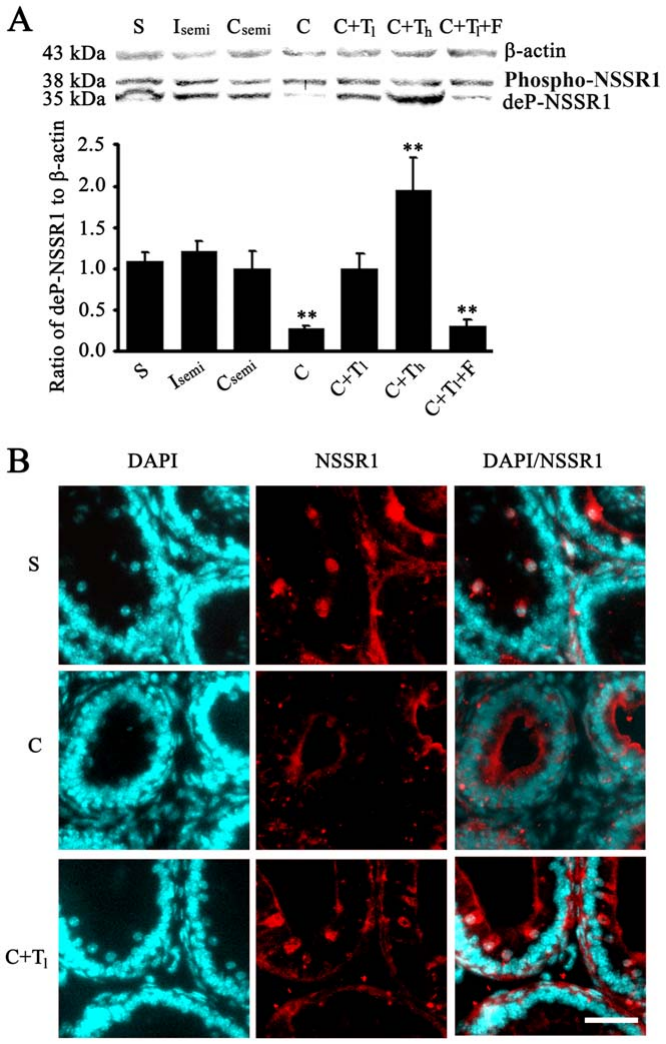
Representative images showing the hormonal regulation of NSSR1 in mouse caput epididymis. A. Western blot analysis of hormonal regulation of NSSR1 in caput epididymis (Representative gels and statistical data). S: Sham operation; Isemi: Intact side of unilateral-castrated mice; Csemi: Castrated side of unilateral-castrated mice; C: Bilateral castration; C+Tl: Injection of low dose testosterone (0.125 mg/day) into bilaterally castrated mice; C+Th: Injection of high dose testosterone (0.5 mg/day) into bilaterally castrated mice; C+ Tl +F: Injection of 0.125 mg testosterone together with 1 mg flutamide per day into bilaterally castrated mice; C+E: Injection of 0.2 mg estradiol into bilaterally castrated mice. ** statistically significant different from sham operated group (P<0.05). B. Representative immunohistological images showing NSSR1 proteins (red) in caput epididymis from sham operated (S), bilaterally castrated (C) and testosterone injected bilaterally castrated (C+ Tl) mice, with nuclear staining (cyan). Bar = 40 µm.

Many genes expressed in the caput epididymis are involved in maintaining the important functions of the epididymis in sperm maturation, and their expressions are usually regulated by testicular factors or hormones [27]. We applied castration surgery and hormone replacement to examine such regulations for NSSR1. The results showed that de-phosphorylated NSSR1 was significantly reduced in the caput epididymis from bilateral-castrated mice but phosphorylated NSSR1 was not, compared to the sham operated mice. In unilateral-castrated mice, the level of both phosphorylated and de-phosphorylated NSSR1 proteins from the castrated-side caput epididymis remained the same as that from the integrated-side (Fig. 3A). These results indicate that NSSR1 might be subjected to regulation by hormones but not testicular factors.

**Figure 4:**
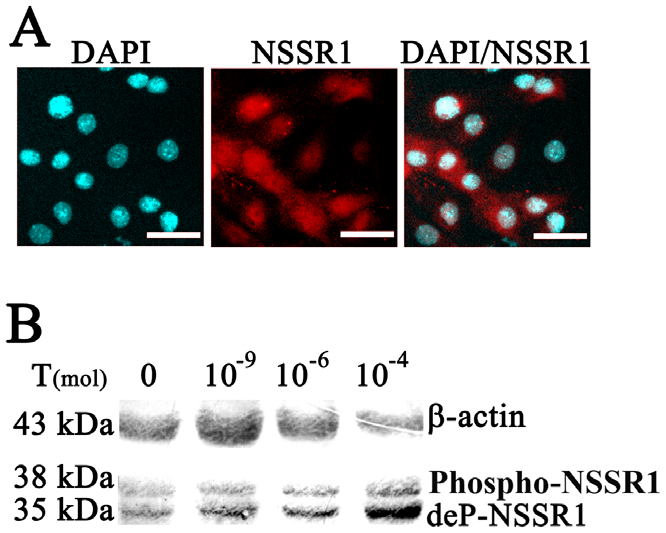
Hormonal regulation of NSSR1 in primarily cultured epididymal epithelial cells. A. Representative IHC images showing the expression of NSSR1 (red) in primarily cultured epididymal epithelium, nuclear were stained with DAPI (presented in cyan). Bars = 40 µm. B. Western blot analysis of hormonal regulation of NSSR1 in primarily cultured epididymal epithelial cells supplemented with 0 nM, 1 nM (10^−9^), 1 µM (10^−6^) and 0.1 mM (10^−4^) testosterone.

To further examine whether NSSR1 is subjected to hormonal regulation, testosterone alone, or testosterone combined with its antagonist flutamide were intraperitoneally injected into the bilaterally castrated mice. NSSR1 expression in the caput epididymis was analyzed by Western blotting and IHC. The results showed that testosterone could restore the expression level of de-phosphorylated NSSR1 in the caput epididymis of the bilateral-castrated mice in a dose dependent manner. In contrast, co-administration of testosterone and flutamide in the bilateral-castrated mice failed to restore the level of de-phosphorylated NSSR1, which remained the same as non-treated bilateral-castrated ones (Fig. 3A). IHC results showed that, in the bilateral-castrated mice, the level of luminal NSSR1 remained unchanged. However, NSSR1 immunostaining in the principle cells decreased to a non-detectable level and the diameter of the lumina of caput epididymal ducts were significantly reduced, which can be restored by application of testosterone (Fig. 3B). The hormonal regulation of NSSR1 in the caput epididymis was confirmed in the primarily cultured epididymis epithelia *in vitro*. The results showed that NSSR1 was expressed in the nucleus and cytoplasm of the primary cultured epididymal cells (Fig. 4A) and the de-phosphorylated NSSR1 was up-regulated by testosterone administration in a dose-dependent manner (Fig. 4B). Taking together, it is indicated that in the caput epididymis, de-phosphorylated NSSR1 proteins, rather than its phosphorylated form, were subjected to hormonal regulation.

### Phosphorylated NSSR1 is localized in the sperm and its subcellular distribution pattern varies in different regions of the epididymis

**Figure 5:**
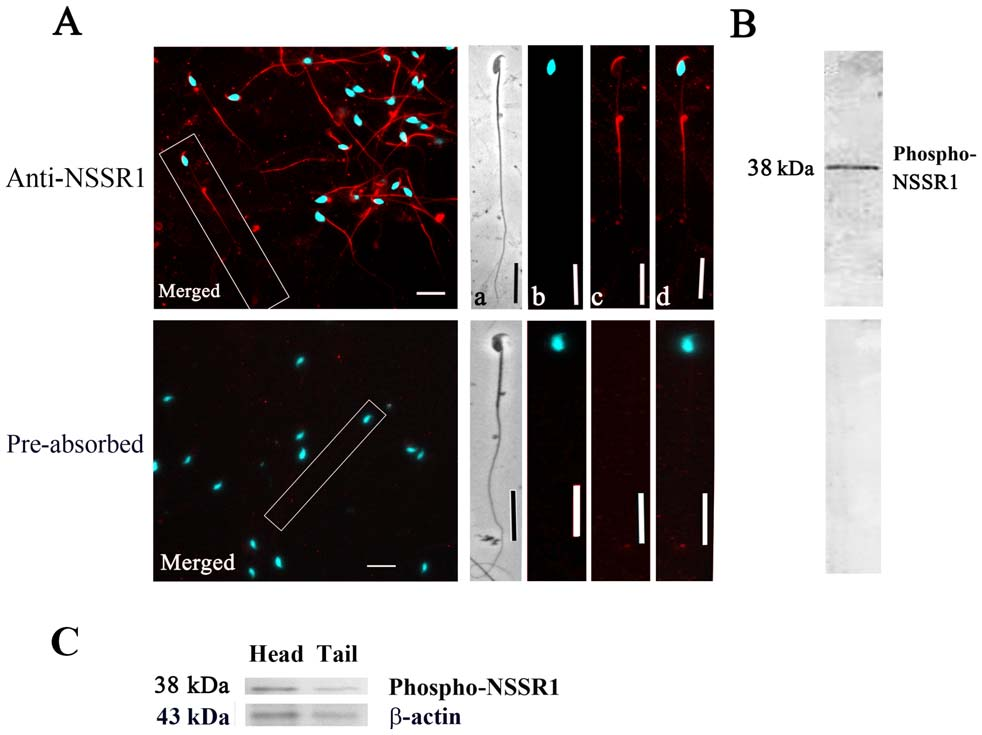
NSSR1 expression in mouse sperm. A. Representative images showing the distribution of NSSR1 proteins (red) in mouse sperms with nuclear stain (cyan). a-d are magnified view of the framed inlet in “Merged” image. Bars = 20 µm. B. Western blot analysis of NSSR1 in sperm lysates. NSSR1 antiserum pre-absorbed with specific NSSR1 peptide was used as the control (Pre-absorbed). C. Western blot analysis of NSSR1 in head and tail of the sperm. β-actin was used as the loading control.

To investigate whether NSSR1 is localized in the sperm, we isolated the sperm from adult mouse epididymis. Immunostaining of NSSR1 was indeed observed in the sperm acrosome and the mid-piece of sperm tail, which was blocked by pre-absorption of the antibody with the specific NSSR1 peptide (Fig. 5A). In consistent, Western blot analysis, using NSSR1 polyclonal antibody, detected a band with molecular weight of 38 kDa corresponding to the phosphorylated NSSR1, which disappeared after pre-absorption of the NSSR1 antibody with the specific NSSR1 peptide (Fig. 5B). In addition, the phosphorylated NSSR1 was also detected in both head and the tail of the sperms by Western blot analysis (Fig. 5C).

**Figure 6:**
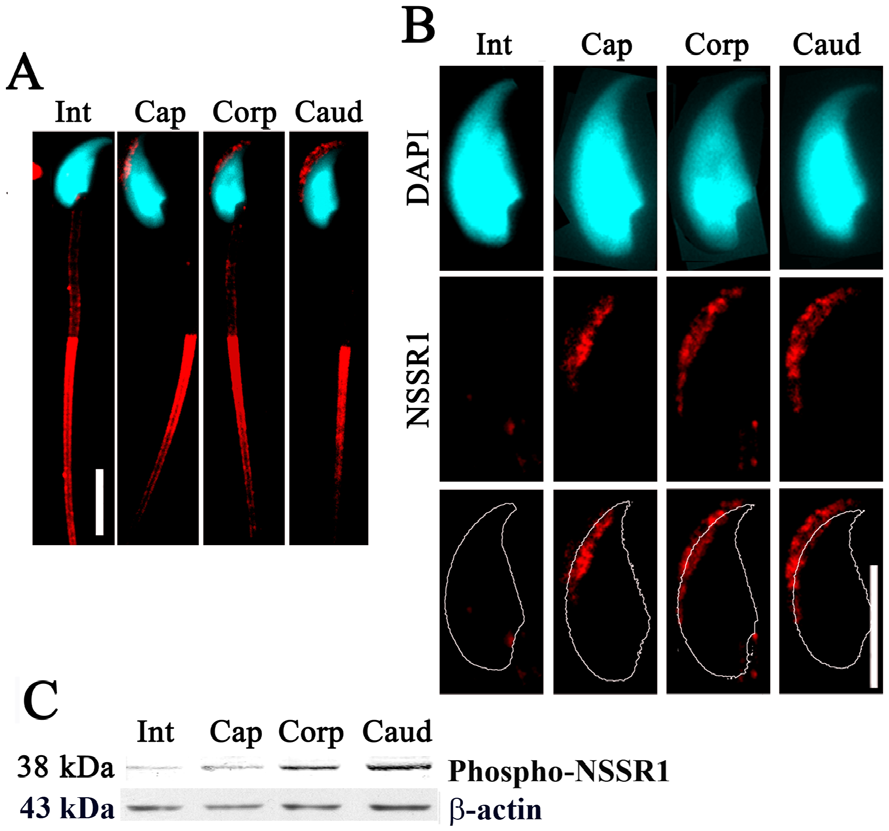
Distribution of NSSR1 in the acrosome of sperms from different regions of mouse epididymis. A. Representative images showing the distribution of NSSR1 (red) in sperms from different regions of mouse epididymis with nuclear stain (cyan). B. Magnified view of the sperm head in A. In the lowest lane of B, the thin gray curves merged with NSSR1 immunostaining (red) are the outlines of sperm heads. Bars = 10 µm. C. Western blot analysis of NSSR1 in sperms from different regions of mouse epididymis. Proteins from equal number of sperms were uploaded. β-actin was used as the loading control. Int: Initial region; Cap: Caput region; Corp: Corpus region; Caud: Cauda region.

We also found that the localization of NSSR1 in the acrosome was changed when the sperm transited through the different regions of epididymis. As shown in Fig. 6, NSSR1 immunostaining was not detected in the acrosome of the sperm from the initial segment of the epididymis, as the sperm transited from the caput to cauda, NSSR1 began to be detectable in the acrosome and the intensity of the staining increased during this process (Fig. 6A, B). Consistently, Western blot analysis of the sperm from different regions of the epididymis showed that the level of NSSR1 increased as the sperm transited from the caput to cauda in the epididymis (Fig. 6C), supporting the results of IHC (Fig. 6B). Moreover, as the sperm transited from the caput to cauda, the distribution of NSSR1 in the acrosome was shown to change from the inner acrosomal region closed to the nucleus to the outer acrosomal region far away from the nucleus (Fig. 6B).

### Decrease of NSSR1 immunostaining in the acrosome of sperms after acrosome reaction

**Figure 7:**
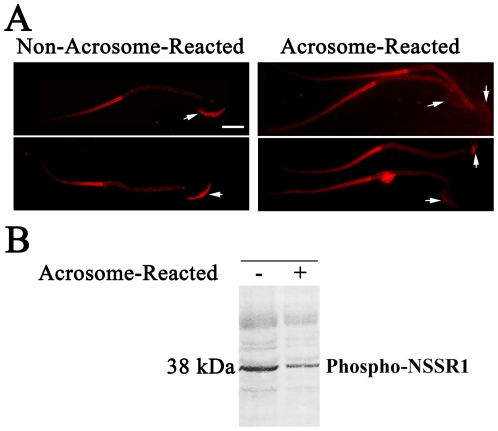
NSSR1 immunostaining in the sperm head disappeared after the acrosome reaction. A. Spermatozoa were washed from disected mouse epididymis with Whitten's Medium and fixed immediately, or capacitated for 2 h in Whitten's medium and for an additional 30 min with the addition of the calcium ionophore A23187, then fixed. Samples were then immunostained with the NSSR1 antibody, followed by FITC-conjugated secondary antibody (presented in red). Arrows indicate the area of the sperm head where the acrosome is located. Bar = 10 µm. B. Western blot analysis of NSSR1 in pellet lysates before and after acrosome reaction.

The finding showing the expression of NSSR1 in the acrosome of cauda sperms raises an interesting question whether NSSR1 expression is regulated by capacitation and acrosome reaction. To address this issue, the cauda sperms collected from mouse epididymis were stained for NSSR1 before or after capacitation and acrosome reaction. The ionophore A23187 was used to ensure completion of the acrosome reaction as previously described [26]. The results showed that NSSR1 immunostaining of the sperm that had undergone capacitation and acrosome reaction reduced significantly compared to that of the uncapacitated one (Fig. 7A), suggesting that NSSR1 proteins were expelled from the sperm head during capacitation and acrosome reaction. Western blot analysis (Fig. 7B) also showed that the 38 kDa phosphorylated NSSR1 decreased significantly after the acrosome reaction, confirming the possibility that NSSR1 proteins were released from the sperm head during capacitation and acrosome reaction.

## Discussion

Spermatozoa released from the testis are immature in that they are immotile and unable to fertilize an oocyte. The epididymis is essential for sperm maturation during sperm transit from testis to vas deferens [28]. In general, the epididymis is subdivided into four major segments designated as initial segment, caput, corpus, and cauda. The principle cells in each region have different functions. Numerous epididymal proteins are regionally expressed and secreted [20]. As sperms move through the epididymis they undergo a series of maturational changes that render them motile and capable of fertilization. Thereby, many biochemical characteristics are modified, such as changes in phospholipid and cholesterol composition of the plasma membrane, modification of plasma membrane protein composition and nuclear condensation of the sperm [28], [29].

Alternative splicing plays roles in sperm maturational changes occurred in epididymis. Several androgen dependent genes that are critical in epididymal function have been shown to be regulated by alternative splicing. For example, the plasma membrane Ca^2+^-ATPase isoform 4 (PMCA4) gene have been demonstrated to express two alternatively spliced isoforms and homozygous mice with a targeted PMCA4 gene deletion are infertile due to severely impaired sperm motility [30]. Cysteine-rich secretory protein-1 (Crisp-1) gene encodes acidic epididymal glycoprotein. There are three splice mRNA variants demonstrated in rat epididymis. Two region-specific forms (D and E) of Crisp-1 protein are secreted into the rat epididymal lumen and binds to sperm heads during their transit through the epididymis [31], [32]. The Pem gene, encoding an atypical homeodomain protein related to Prd/Pax family members, produces an epididymis-specific mRNA isoform via alternative splicing mechanism in the presence of androgen [33]. In contrast to the studies described above, the molecular mechanism underlying the splicing of these genes has rarely been studied. SR protein, a family of proteins with a characteristic domain rich in arginine and serine residues (RS domain), are important regulators of the alternative splicing [34]. To our knowledge, the present study is for the first time to analyze the expression of a SR protein in the epididymis.

In the present study, we noticed that NSSR1 in mouse caput is largely phosphorylated and localized in the apical side of epithelial cells. During the development, de-phosphorylated NSSR1 in the caput is up-regulated, which is in concert with the increased NSSR1 immunofluorescence signal observed in the principle cells. We also noticed that, in the castration and hormone replacement experiments, the expression level of de-phosphorylated NSSR1, rather than phosphorylated NSSR1, is significantly deceased in mouse epididymis after castration. De-phosphorylated NSSR1 has been previously shown to act as a splicing repressor and phosphorylated NSSR1 act as a splicing activator [11], [12], [13]. Our results suggest an interesting possibility that testosterone may repress tissue-specific alternative mRNA splicing in the epididymis by regulating the level of de-phosphorylate NSSR1. It has been demonstrated that a subset of SR proteins shuttles between the nucleus and the cytoplasm based upon their phosphorylation status [35]. Interestingly, we found that in adult epididymis caput, NSSR1 is mainly distributed in cytoplasm of the cells, but in primary cultured epididymal epithelial cells, NSSR1 is expressed in both nuclear and cytoplasm, hinting that it may also shuttle between the nucleus and the cytoplasm. In our previous studies, we also showed that phosphorylation status of NSSR1 is related to its sub-cellular distribution in mouse testes [18]. Hence,it is also possible that hormone may affect NSSR1 subcellular distribution in mouse epididymis by regulating the phosphorylation status.

The observation that phosphorylated NSSR1 is highly expressed in the acrosome of cauda sperm, and is released from the sperm during the acrosome reaction, suggests that NSSR1 may play a role in the fertilization process. The acrosome contains hydrolytic enzymes that are necessary for fertilizing the egg. Capacitation occurs after sperm ejaculation and is necessary for the acrosome reaction to occur. Only after the acrosome reaction are the sperms competent to fertilize an oocyte [36], [37]. Previous studies have shown that a lot of important genes assiociated with capacitation or acrosome reaction undergo alternative splicing in acrosome such as CD46 and Sarcoplasmic/-endoplasmic reticulum Ca2+-ATPases 2(SERCA2) [38], [39]. However,as it is not likely that NSSR1 regulates splicing in acrosome or sperm tail, some other roles may be played by the NSSR1 proteins in these sites. A subset of SR proteins such as SF2/ASF was found to shuttle between nucleus and cytoplasm and be able to participate in the export of spliced mRNAs, nonsense-mediated mRNA decay and translation of certain mRNAs [34]. The expression of NSSR1 in acrosome and sperm tail but not nucleus may also hint that it may play roles in these functions. It will be extremely interesting to further address the questions regarding how NSSR1 involves in capacitation and acrosome reaction.

In summary, we demonstrated the developmental regulation and cellular distribution of NSSR1 in mammalian epididymis. Both de-phosphorylated and phosphorylated NSSR1 are expressed in the caput, while no phosphorylated NSSR1 is expressed in the corpus and cauda. The expression of dephosphorylated NSSR1 increased significantly during development of the caput epididymis. In adult mice, we found that the phosphorylated NSSR1 was expressed in the apical side of epithelial cells, and dephosphorylated NSSR1 in caput epididymis was increased in a testosterone dependent manner. In addition, phosphorylated NSSR1 is also found to be abundantly expressed in the mid-piece of the sperm tail as well as sperm acrosome. The distribution of NSSR1 in the sperm head varies within the four regions of the epididymis. Furthermore, NSSR1 immunostaining in the sperm head decreased after the capacitation and acrosome reaction. This work for the first time describes an androgen dependent expression of splicing factors in the epididymis, and provides a foundation for further understanding the role of NSSR1 in sperm maturation and fertilization.

## References

[1] Black DL (2000). Protein diversity from alternative splicing: a challenge for bioinformatics and post-genome biology.. Cell.

[2] Johnson JM, Castle J, Garrett-Engele P, Kan Z, Loerch PM (2003). Genome-wide survey of human alternative pre-mRNA splicing with exon junction microarrays.. Science.

[3] Smith CW, Valcarcel J (2000). Alternative pre-mRNA splicing: the logic of combinatorial control.. Trends Biochem Sci.

[4] Jurica MS, Moore MJ (2003). Pre-mRNA splicing: awash in a sea of proteins.. Mol Cell.

[5] Lopez AJ (1998). Alternative splicing of pre-mRNA: developmental consequences and mechanisms of regulation.. Annu Rev Genet.

[6] Konig H, Matter N, Bader R, Thiele W, Muller F (2007). Splicing segregation: the minor spliceosome acts outside the nucleus and controls cell proliferation.. Cell.

[7] Hastings ML, Krainer AR (2001). Functions of SR proteins in the U12-dependent AT-AC pre-mRNA splicing pathway.. Rna.

[8] Steitz JA, Dreyfuss G, Krainer AR, Lamond AI, Matera AG (2008). Where in the cell is the minor spliceosome?. Proc Natl Acad Sci U S A.

[9] Feng Y, Valley MT, Lazar J, Yang AL, Bronson RT (2009). SRp38 regulates alternative splicing and is required for Ca(2+) handling in the embryonic heart.. Dev Cell.

[10] Komatsu M, Kominami E, Arahata K, Tsukahara T (1999). Cloning and characterization of two neural-salient serine/arginine-rich (NSSR) proteins involved in the regulation of alternative splicing in neurones.. Genes Cells.

[11] Shin C, Feng Y, Manley JL (2004). Dephosphorylated SRp38 acts as a splicing repressor in response to heat shock.. Nature.

[12] Shin C, Manley JL (2002). The SR protein SRp38 represses splicing in M phase cells.. Cell.

[13] Feng Y, Chen M, Manley JL (2008). Phosphorylation switches the general splicing repressor SRp38 to a sequence-specific activator.. Nat Struct Mol Biol.

[14] Ling IF, Estus S Role of SFRS13A in low-density lipoprotein receptor splicing.. Hum Mutat.

[15] Liu L, Chen XH, Huang J, Lin JJ, Lin WM (2004). NSSR1 promotes neuronal differentiation of mouse embryonic carcinoma P19 cells.. Neuroreport.

[16] Peng ZY, Lee SC, Chen XH (2007). The expression and distribution of neural salient serine/arginine-rich protein 1 in rat retina.. Neuroreport.

[17] Liu L, Lin JJ, Chen X, Liu X, Xu P (2003). Neural expression and regulation of NSSR1 proteins.. Neuroreport.

[18] Xiao PJ, Hu L, Li J, Lin W, Chen X (2007). NSSR1 is regulated in testes development and cryptorchidism and promotes the exon 5-included splicing of CREB transcripts.. Mol Reprod Dev.

[19] Peng ZY, Xiao PJ, Qi Y, Zhang W, Chen XH NSSR1 is regulated by testosterone in the mouse uterus and extensively expressed in endometrial carcinoma.. Tumour Biol.

[20] Cornwall GA (2009). New insights into epididymal biology and function.. Hum Reprod Update.

[21] Peng ZY, Huang J, Lee SC, Shi YL, Chen XH (2009). The expression pattern of heterogeneous nuclear ribonucleoprotein R in rat retina.. Neurochem Res.

[22] de las Heras MA, Calandra RS (1987). Androgen-dependence of ornithine decarboxylase in the rat epididymis.. J Reprod Fertil.

[23] Wennemuth G, Carlson AE, Harper AJ, Babcock DF (2003). Bicarbonate actions on flagellar and Ca2+ -channel responses: initial events in sperm activation.. Development.

[24] Furland NE, Oresti GM, Antollini SS, Venturino A, Maldonado EN (2007). Very long-chain polyunsaturated fatty acids are the major acyl groups of sphingomyelins and ceramides in the head of mammalian spermatozoa.. J Biol Chem.

[25] Whitten WK, Biggers JD (1968). Complete development in vitro of the pre-implantation stages of the mouse in a simple chemically defined medium.. J Reprod Fertil.

[26] Talbot P, Summers RG, Hylander BL, Keough EM, Franklin LE (1976). The role of calcium in the acrosome reaction: an analysis using ionophore A23187.. J Exp Zool.

[27] Chauvin TR, Griswold MD (2004). Androgen-regulated genes in the murine epididymis.. Biol Reprod.

[28] Cooper TG (1996). Epididymis and sperm function.. Andrologia.

[29] Cherry J, Karschner V, Jones H, Pekala PH (2006). HuR, an RNA-binding protein, involved in the control of cellular differentiation.. In Vivo.

[30] Wilhelm B, Brandenburger T, Post H, Aumuller G (2008). Expression and localization of PMCA4 in rat testis and epididymis.. Histochem Cell Biol.

[31] Roberts KP, Ensrud KM, Wooters JL, Nolan MA, Johnston DS (2006). Epididymal secreted protein Crisp-1 and sperm function.. Mol Cell Endocrinol.

[32] Roberts KP, Hoffman LB, Ensrud KM, Hamilton DW (2001). Expression of crisp-1 mRNA splice variants in the rat epididymis, and comparative analysis of the rat and mouse crisp-1 gene regulatory regions.. J Androl.

[33] Clement JQ, Maiti S, Wilkinson MF (2001). Localization and stability of introns spliced from the Pem homeobox gene.. J Biol Chem.

[34] Long JC, Caceres JF (2009). The SR protein family of splicing factors: master regulators of gene expression.. Biochem J.

[35] Sanford JR, Ellis JD, Cazalla D, Caceres JF (2005). Reversible phosphorylation differentially affects nuclear and cytoplasmic functions of splicing factor 2/alternative splicing factor.. Proc Natl Acad Sci U S A.

[36] Luconi M, Krausz C, Forti G, Baldi E (1996). Extracellular calcium negatively modulates tyrosine phosphorylation and tyrosine kinase activity during capacitation of human spermatozoa.. Biol Reprod.

[37] Visconti PE, Bailey JL, Moore GD, Pan D, Olds-Clarke P (1995). Capacitation of mouse spermatozoa. I. Correlation between the capacitation state and protein tyrosine phosphorylation.. Development.

[38] Johnson PM, Clift LE, Andrlikova P, Jursova M, Flanagan BF (2007). Rapid sperm acrosome reaction in the absence of acrosomal CD46 expression in promiscuous field mice (Apodemus).. Reproduction.

[39] Lawson C, Dorval V, Goupil S, Leclerc P (2007). Identification and localisation of SERCA 2 isoforms in mammalian sperm.. Mol Hum Reprod.

